# Functional Properties and Antioxidant Activity of *Morus alba* L. Leaves var. Zolwinska Wielkolistna (WML-P)—The Effect of Controlled Conditioning Process

**DOI:** 10.3390/antiox9080668

**Published:** 2020-07-26

**Authors:** Monika Przeor, Ewa Flaczyk, Dominik Kmiecik, Maciej S. Buchowski, Halina Staniek, Aneta Tomczak-Graczyk, Joanna Kobus-Cisowska, Anna Gramza-Michałowska, Joanna Foksowicz-Flaczyk

**Affiliations:** 1 Department of Gastronomy Science and Functional Foods, Faculty of Food Science and Nutrition, Poznan University of Life Sciences, Wojska Polskiego 31, 60-624 Poznan, Poland; ewa.flaczyk@up.poznan.pl (E.F.); dominik.kmiecik@up.poznan.pl (D.K.); joanna.kobus-cisowska@up.poznan.pl (J.K.-C.); anna.gramza@up.poznan.pl (A.G.-M.); 2 Division of Gastroenterology, Hepatology and Nutrition, Department of Medicine, Vanderbilt University, Nashville, TN 37072, USA; maciej.buchowski@vumc.org; 3 Department of Bromatology and Food Toxicology, Institute of Human Nutrition and Dietetics, Faculty of Food Science and Nutrition, Poznan University of Life Sciences, Wojska Polskiego 31, 60-624 Poznan, Poland; halina.staniek@up.poznan.pl; 4 Department of Food Biochemistry and Analysis, Faculty of Food Science and Nutrition, Poznan University of Life Sciences, Mazowiecka 28, 60-623 Poznan, Poland; aneta.graczyk@up.poznan.pl; 5 Institute of Natural Fibres and Medicinal Plants, Wojska Polskiego 71 B, 60-630 Poznan, Poland; joanna.flaczyk@iwnirz.pl

**Keywords:** *Morus alba*, antioxidant activity, conditioning, phenolic acids, flavonols, DNJ, polyphenols, semi-technical processing, ABTS test, DPPH test

## Abstract

This study evaluated the effect of adding a new step, termed conditioning, to the traditional processing of leaves from *Morus alba* var. zolwinska wielkolistna grown in Poland (WML-P). This step, modeled on tea leaves processing, was conducted in a controlled environment on a semi-technical scale. The primary goal was to evaluate the effect of the WML-P conditioning for 1–4 h at 32–35 °C on the content of bioactive compounds (total phenolics, phenolic acids, flavonols, 1-deoxynojirimycin) and antioxidant activity (radical scavenging against DPPH, antioxidant capacity, chelating activity and ferric reducing antioxidant potential) of the lyophilized extracts. For the first time WML-P extracts content was comprehensively characterized by assessing dietary fiber fractions, fatty acids, amino acids, macro- and microelements and chlorophyll content. Compared to the traditional process, adding the conditioning step to WML-P processing resulted in an increased total phenolics content, radical scavenging capacity, ability to quench 2,2-diphenyl-1-picrylhydrazyl (DPPH^•^) and iron-chelating ability in the lyophilized extracts. The beneficial effect depended on conditioning time. The highest flavonols and phenolic acids content were found after 2-h conditioning. We concluded that adding a 2-h conditioning step to traditional WML-P processing results in getting WML-P lyophilized extract with increased bioactive compounds content and high antioxidant activity.

## 1. Introduction

There are about 30 species of mulberry trees in the world, half of which are native to China. White mulberry (*Morus alba* L., Folium Mori) (WM) belongs to the Moraceae family [1]. In central Poland, a Polish variety of WM, var. zolwinska wielkolistna (WML-P) is cultivated. Typically, only fruits and leaves from the mulberry tree are utilized due to the ease of harvesting and low acquisition costs.

Traditionally, *Morus alba*, as other *Morus* species, has been utilized in Chinese, Japanese, Indian and Korean traditional medicine. Mulberry products have been used to nourish blood and benefit kidneys and as a treatment for weakness and fatigue, urinary incontinence, dizziness, tinnitus, constipation and in more recent years, are considered helpful in the treatment of some cancers [2]. Over time, mulberry products have also been used as a food component. Compared with other morphological parts, fruits are the most popular [3] and white mulberry leaves (WML) show the highest antioxidant activity.

It has been reported that antioxidants present in the WML products could potentially balance free radicals and protect against their destructive effects on genetic material and physiological processes.

Numerous reports from animal studies have documented strong antidiabetic properties of the WML [4]. In human studies, consumption of food products enriched with the WML extract added into diet resulted in lower glycemic increase compared to non-enriched products in both, patients with diabetes type 2 and healthy controls and reduced excessive body weight [5]. The compound responsible for the lowering of blood glucose concentration is 1-deoxynojirimycin (DNJ) and its derivatives, which are inhibitors of intestinal α-glucosidase.

WML products consumed with a diet are thought to protect against liver cancer, to prevent obesity-related non-alcoholic fatty liver, hypolipidemic or lipid-regulating properties in blood protect against atherosclerosis, to show anti-aggregative properties in platelet and anti-inflammatory effects or to reduce anxiety [6,7].

It has been reported that some medicinal plants show a neuroprotective effect [8]. For example, extracts from young leaves and mulberry shoots rich in oxyresveratrol are utilized in the treatment of neurodegenerative diseases. Most studies, did not find the toxic effects of WML preparations. Reported beneficial health and therapeutic properties of WML products are most likely associated with the presence of bioactive compounds, including flavonoids, phenolic acids, iminosugars, tannins, polysaccharides and alkaloids [9]

In many countries, WML products are commonly used as drought or tea infusion with a specific tonic, low toxicity and excellent therapeutic properties. Currently, many new technologies use WML products as a component of functional foods, often based on recipes derived from folk cuisine. Mulberry products are also used as supplements of traditional foods, such as dairy, bakery, bread and snacks [10,11].

In recent years, the mulberry tree is experiencing an increase in reproduction and cultivation, progress in pest management and finding or confirming the therapeutic properties of WML components [1]. The WML products on the market include a variety of teas, similar to ‘regular’ yellow, red or black tea made from *Camellia sinensis*, which very often use conditioning as part of processing [12]. To best of our knowledge, there are no studies, reporting if conditioning of WML would affects characteristics, properties and antioxidant capacity of WML products.

The WML processing in practice usually involves minimal processing but differences and especially drying and shredded temperatures affect the chemical composition, antioxidative properties and potential health benefits of the product [13].

Most of the currently available data relate to Asian raw material and do not include the description of mulberry leaves preparation. In one study, Chon et al. [14] used the fermentation step in the processing of Korean WML leaves form Korean trees. In their study, a methanol extract from fermented leaves showed higher than unfermented leaves antiproliferative activity in the human stomach and colon cancer cells. Although the study may indicate that increased antioxidant properties of fermented extract. The study did not report the effect of fermentation on changes in the antioxidant activity if the extracts.

In previous work, we showed that differences in WML-P antioxidant activity depend on drying temperatures [13] and the WML-P extracts included in diet reduce hyperglycemia in diabetic rats [15]. However, we did not use leaves conditioning process or assess the content of antioxidants or certain compounds with certain potential health benefits in the dried extracts.

Therefore, this study aimed to investigate if adding of processing step termed conditioning to traditional WML-P processing would affect chemical composition and characteristics of the lyophilized extracts and improve its nutritional quality and functional properties, including antioxidant capacity.

Our primary hypothesis was that WML-P conditioning will cause an increase in the antioxidant capacity of the lyophilized extract. Our secondary hypothesis was that changes in the functional properties of WML-P will depend on conditioning time.

## 2. Material and Methods

### 2.1. Plant Material

#### 2.1.1. Conditioning

Fresh white mulberry (*Morus alba* L.) leaves form Polish var. zolwinska wielkolistna (WML-P) were picked manually at the mulberry tree farm in Pętkowo (Institute of Natural Fibres & Medicinal Plants), near Poznan, central Poland, in June and July 2011.

**Figure 1:**
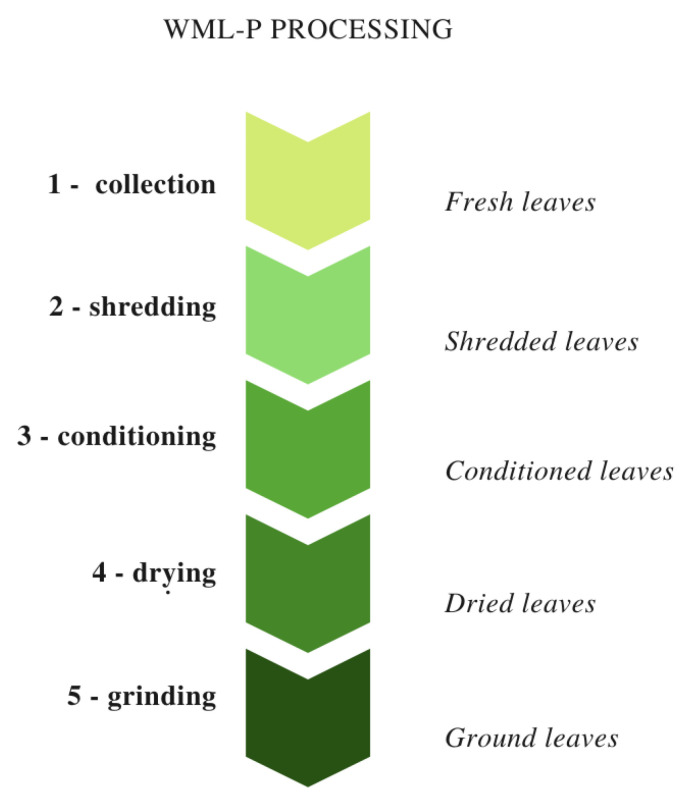
Technological processing of var. zolwinska wielkolistna (WML-P), including controlled conditioning, a novel step in traditional *Morus alba* leaf processing.

Technological processing of WML-P was conducted at the Institute of Agricultural and Food Biotechnology in Poznan, Poland (IAFB) (Figure 1). The raw leaves were shredded using a chopper/crusher (Stihl, Viking GE 103, Tengen, Germany) into particles measuring approximately 50–70 mm in length and 10–20 mm in width, divided into 5 batches and placed on wooden sieve trays in prisms (100 cm long, 40 cm wide, 10–15 cm high). Leaves from on one tray were not conditioned and used as a control sample. The remaining 4 trays were conditioned in the industrial dryer (SSO, Izoterma, Łany, Poland) for 1 h, 2 h, 3 h or 4h at 32.0 °C ± 3.0. Each of WML-P samples was air-dried in the tunnel dryer (IAFB, Poznań, Poland) at inlet temperature 90 °C and outlet temperature 60 °C. Dried WML-P from each batch were grounded (400 rpm for 15 s) in the laboratory mill (Retsch, GM200, Haan, Germany) to a powder (0.8–0.08 mm) and stored in polyethylene bags at room temperature for processing and analyzes.

#### 2.1.2. Extraction Process

Aqueous extracts were prepared from 10.0 g ± 0.2 of shredded WML-P. Each WML-P sample was extracted two times by mixing with boiling distilled water (100 mL and 40 mL) and heat for 15 min in 100 °C (EM, Thermo Scientific, Waltham, MA, USA). The extract was separated from the sediment and filtered using the Büchner funnel and Whatman filter paper (No.1, 1–11 µm). Filtered samples were lyophilized (Christ Alpha 1–4 LSC, *p* < 1 mBa, capacitor temperature −50 °C, shelf temperature 20 °C) and the dried extracts stored in plastic bags (in refrigerator at 4.0 °C) for further analyzes.

### 2.2. Chemical Composition

#### 2.2.1. Basic Chemical Composition

Total protein, fat, ash and dry matter contents were determined the AOAC methods [16]. The total carbohydrates content (g/100 g) was calculated using the equation:

(1) total carbohydrates=100−(protein+crude fat+ash).

#### 2.2.2. Dietary Fiber Fractions

The contents of total dietary fiber (TDF), soluble dietary fiber (SDF) and insoluble dietary fiber (IDF) were measured using Fibertec System E 1023 (Foss Tecator, Hilleroed, Sweden). The content of neutral dietary fiber (NDF), acid detergent fiber (ADF), acid detergent lignin (ADL) and cellulose (C) was determined using the detergent [17]. The content of hemicellulose (H) was calculated as follows:

(2) H=NDF−ADF

Analyses were conducted using the Fibertec System M 1020 (Foss Tecator, Hilleroed, Sweden).

#### 2.2.3. Fatty Acids (FA) Composition

The fatty acids composition was determined according to the standard methodology [18] using Agilent 7820A GC (Agilent Technologies, Santa Clara, CA, USA) equipped with a flame ionization detector (FID) and SP-2560 (100 m, 0.25 mm, 0.20 µm) column (Supelco, Bellefonte, PA, USA). The FA methyl esters (FAME) were separated under the following conditions—the initial oven temperature 150 °C and increased to 200 °C at 1.5 °C/min; the injector and detector temperatures were 250 °C; split 1:10; injection volume 0.5 µL and the helium at flow rate 1 mL/min was used as carrier gas. The FAME were identified by comparing with commercially available standards (Grain Fatty Acid Methyl Ester Mix, Supelco, Bellefonte, PA, USA). The results were expressed as % of total FA [19].

#### 2.2.4. Amino Acids (AA)

The AA content was determined with HPLC (Nexera X2 Shimadzu LC-30AD, Kyoto, Japan) equipped with a DAD detector (SPD-M30A, Shimadzu, Kyoto, Japan), using Waters column (ACCQ-TAG Ultra C18, 100 mm, 2.1 mm, 1.7 µm) at λ = 260 nm). First, samples were hydrolyzed according to AOAC methods [20,21]. The AA were separated under the following conditions—the injection volume was 1 µL; flow rate 0.6 mL/min; separation temperature 55 °C; total method time 22 min; separation time 18 min; the mobile phases were based on C_2_H_3_N (ready-to-use Waters eluents) as 100% solution of AssQ-Taq Ultra (eluent A) and 5% solution of AssQ-Taq Ultra (eluent B); elution was initiated at 100% of eluent A and 0% eluent B. Non-linear gradient separation was a gradual increase in the ratio of phase B to phase A. The level of eluent B increased to a maximum of 59.6% and then decreased to 0%. Qualitative and quantitative analyses were conducted by comparison with commercially available standards (Waters, Elstree, UK) dissolved in water. The results were expressed as g/100 g.

#### 2.2.5. Macro- and Microelements

The atomic absorption spectrometer equipped with a flame atomizer (AAS-3, Carl-Zeiss Jena, Jena, Germany) was used to determine contents of magnesium (Mg), calcium (Ca), manganese (Mn), iron (Fe), copper (Cu), zinc (Zn), sodium (Na) and potassium (K). The WML-P samples were mineralized in a microwave oven (Mars-5, CEM Co., Charlotte, NC, USA) with nitric acid (65%) [22]. The results were expressed in µg/g or mg/g of dried leaves. Certificated materials Soy Bean Flour INCT-SBF-4 and Bovine Liver-Trace Elements NIST-1577 c (Institute of Nuclear Chemistry and Technology in Warsaw, Poland) were used as reference standards.

#### 2.2.6. Chlorophyll a and b

The contents of chlorophyll a and b were determined using the Eder method [23]. Absorbance in the methanol fraction was measured using a Metertech SP-830 PLUS (Nangang, Taipei, Taiwan) spectrophotometer at a wavelength of 645 nm (chlorophyll b) and 663 nm (chlorophyll a).

#### 2.2.7. Phenolic Acids and Flavonols

Phenolic acids: gallic acid (GAL), protocatechuic acid (PRO), 4-hydroxybenzoic acid (HYD), vanillic acid (VAN), caffeic acid (CAF), chlorogenic acid (CHL), *p*-coumaric acid (CUM), ferullic acid (FER), sinapic acid (SIN) and flavonols: rutin (RUT), 3-β-glucoside (isoquercitrin) (ISQ), quercetin 3-*O*-(6”-*O*-malonyl)-β-d-glucoside (MAL), kaempferol 3-glucoside (astragalin) (AST), myricetin (MYR), quercetin (QUE), kaempferol (KEM), isorhamnetin (ISR) contents were determined using HPLC/DAD (Agilent Infinity 1290, CA, USA). A filtered (0.45 µm PTFE filters, Millipore) sample (20 µL) was injected into the thermostated C18 Zorbax SB column (15 mm × 3.9 mm I.D., 5 µm; Agilent Technology, Santa Clara, CA, USA) according to a method in References [24,25] with our own modifications. The mobile phases were H_3_PO_4_ (pH = 2.7) (solvent A) and C_2_H_3_N (50%) (solvent B) at a flow rate of 1.0 mL/min (average flow rate 1.99 min, ranging from 6.10 to 76.00 min) and 0.6 mL/min (from 2.00 to 6.00). Elution was initiated at 90% of solvent A, maintained for 27 min and then decreased to 60% in 29 min and maintained for 5 min and then decreased to 56% in 2 min and maintained for 8 min. Finally, starting at 71 min, the concentration of solvent A was increased to 90% in 1 min and maintained until 76 min. Chromatograms were recorded at a wavelength of 260 nm, 310 nm, 370 nm. The identification of the compounds was performed by comparing the retention times and photodiode array spectra with those obtained for reference standards. Quantification of identified phenolic acids and flavonols was made by comparing the area under the peaks with those of the standard (Sigma-Aldrich, St. Louis, MO, USA). Accuracy of the method in our laboratory was 96.99–101.95%, limit of detection (LOD) was 3.3 ng/mL and the limit of quantification (LOQ) was 10.0 ng/mL.

#### 2.2.8. Total Phenolics

Total phenolics content in the dried WML-P was determined using the Folin-Ciocalteu colorimetric method [26]. Results were expressed as gallic acid equivalent (mg GAE/g of dried leaves).

#### 2.2.9. 1-Deoxynojirimycin (DNJ)

High-performance liquid chromatography with fluorescence detector (Ex: 254 nm, Em: 322 nm) (Agilent Infinity 1260, Santa Clara, CA, USA) was used to determine the content of DNJ. The separation was done with TSKgel Tosoh Amide-80 (250 mm × 4.6 mm ID × 5 µm) column (Tosoh Bioscience, Tokyo, Japan), according to Kim et al. [27] with slight modifications. The sample (200 mg) was mixed with HCl (1 mL, 0.05 M), vortexed and centrifuged (15 min, Eppendorf Centrifuge 5702R, 4400 rpm). The supernatant was separated and 10 µL sample was mixed with potassium borate buffer (0.4 M, pH 8.5, 10 µL), mixed with 2 µL of FMOC-Cl (5 mM) in 1.5 mL microtube and allowed to react at 20 °C for 20 min in a water circulator. The reaction was terminated by adding 10 µL of glycine (0.1 M) and the mixture was diluted with 968 µL of CH_3_COOH (0.1%) and filtered through 0.45 µm syringe PTFE filter (Millipore). The sample (5 µL) was injected into the thermostated (30 °C ± 0.8) column by the thermostated autosampler (30 °C ± 1.0). The mobile phases were methanol (eluent A) and C_2_H_3_N with CH_3_COOH (0.1%) (*v/v*, 1:1) (eluent B) at flow rate of 1.0 mL/min from 0.00 min to 2.00 min, 0.5 mL/min from 2.00 min to 4.50 min and 1.2 mL/min from 7.00 min to 10.00 min. The elution was initiated with 55% of eluent A, increased to 75% for 2 min, maintained for 2.50 min, decreased to 55% eluent A in 2.10 min and maintained for the next 3 min. DNJ was identified by comparing the retention times and spectra with methanol-diluted reference standard and quantified by comparing the area under the peak to the standard. The accuracy of the method was 97.95% and the limit of detection (LOD) and quantification (LOQ) were 6.7 ng/mL and 20.0 ng/mL, respectively

### 2.3. Antioxidant Activity

#### 2.3.1. Radical Scavenging Capacity Against DPPH

The free-radical scavenging potential of WML-P extracts was measured in the methanol solution of DPPH^•^ (2,2-diphenyl-1-picrylhydrazyl) according to a method described by Amarowicz et al. [28]. Results were expressed as Trolox (TE) equivalent (µM TE/g of dried leaves).

#### 2.3.2. Total Antioxidant Capacity with ABTS^•+^

The method was based on the SET (single electron transfer) mechanism, according to Re et al. [29] method and expressed as µM TE/g of dried leaves.

#### 2.3.3. Chelating Activity

The chelating activity of WML-P extracts was determined using a method described by Tang et al. [30]. Deionized water was used as a control and ferrozine as a reference standard. The chelating activity was calculated as follows:

(3) $$1-\left[\left({\mathrm{Abs}}_{\mathrm{sample}}-{\mathrm{Abs}}_{\mathrm{reference}}\right)÷{\mathrm{Abs}}_{\mathrm{control}}\right]\times 100$$

and expressed as a percentage (%) of reference standard activity.

#### 2.3.4. Ferric Reducing Antioxidant Potential (FRAP) Assay

FRAP assay was conducted using Benzie and Strain [31] method. The results were expressed as FeCl_3_ equivalent (µM Fe^2+^) per mg of dried leaves.

### 2.4. Reagents

All the chemicals were of analytical grade or chromatographic grade, purchased from POCH, Gliwice, Poland or Merck, Darmstadt, Germany.

### 2.5. Statistical Analysis

All experiments were performed in triplicate and the results are mean ± SD. The amino acid content of WML-P analysis was performed in duplicate. Data were analyzed using one-way analysis of variance (ANOVA), followed by Tukey’s post-hoc test. Statistical differences were calculated at the significance level *p* < 0.05 and represented by superscript letters. The statistical analysis was performed using Statistica software, version 13 (StatSoft, Kraków, Poland).

## 3. Results and Discussion

Similarly to WML cultivated in China, Korea, India, Japan and Thailand, WML-P extracts have excellent nutritional quality [32,33] and have been used in the prevention and/or treatment of chronic diseases, in particular diabetes [15]. This study reports the effects of a new step termed conditioning added to traditional WML-P processing on chemical composition, the content of bioactive components and antioxidative activity in the lyophilized WML-P extracts for the first time. This process simulated withering, fermentation and fixation processes used in tea productions. The conditioning parameters were previously tested in our laboratory and adapted to a semi-technical scale [13].

### 3.1. Chemical Composition

Dried WML-P contained approximately 99% of dry matter, 11–13% of protein, 1–2% of fat and 2–16% of ash (Table 1A). Noteworthy is protein content in the WML-P was 2–6% lower than the content found in Pakistan WML [34]. Conditioning for up to 3 h did not reduce the protein content of extract. This finding is of practical importance because in other studies [35], the WML were used as protein supplements replacing oilseed meal in the diet of mono-gastric animals. This might suggest that mulberry foliage could be also used as a diet component for farm animals.

The highest amount of AA (24.676 g/100 g), including EAA (8.805 g/100 g) were found in the control sample. During conditioning, AA content decreased by 23.1% (1 h), 29.9% (2 h), 25.7% (3 h), 37.6% (4 h) (Table 1B). The ratio of EAA to NEAA was ranging from 65% to 67% and was similar to the ratio in Chinese WML reported by Yao et al. [35]. Compared to Chinese WML, the WML-P contained more Gly, Lys, Val, Phe, Ile, Arg and Thr [35].

The total fat content was 1.02–1.79% in WML-P. Among FA, combined MUFA and PUFA contributed to almost ⅔ of all FA (Table 1C). Among PUFAs, α-linolenic acid accounted for almost half and linoleic acid for ~10% of total FA, indicating that the FA profile in WML-P is nutritionally beneficial. The UFA/SFA ratio regardless of the conditioning time, was ~1.9–2.0. This ratio was higher than reported by Radojkovic et al. in Serbian WML [36]. FA are generally well tested in seeds and oils [37]. Sánchez-Salcedo et al. [38] analyzed the FA profile in *Morus alba* fruits and found the higher content of linoleic acid than α-linolenic acid.

Of note, both the AA composition and FA profile of WML-P are reported for the first time.

The minerals content of WML-P is in Table 1D. Among minerals, Ca and Mg were present in the highest amounts and were similar to those found in Thai and Serbian WML [39,40]. Content of Fe and Mn, cofactors of many enzymes, including catalase in photosynthetic cells, did not change after conditioning.

The dietary fiber content in conditioned WML-P samples was 4–5% higher than in control (Table 1E). The dominant fraction was IDF (92%). Compared to our study, Srivastava et al. [41] found in Indian WML about 1–5% more total fiber but a similar amount of NDF. The NDF is important in maintaining a balanced composition of human intestinal microflora. The amount of fiber in WML-P was comparable to those in other leafy vegetables such as dill, parsley and cabbage. The lignin content in WML-P increased during conditioning from 0.10 to 0.18 g/100 g). It is possible is the increase was caused by lignin’s ability to bind polyphenols to indeterminable complexes with lower antioxidant activity but it requires future studies.

The ratio of chlorophyll a to b was 3:2 and did not change after conditioning. Crushing tissue during the initial steps of WML-P processing most likely caused tissue maceration of both control and conditioned samples. Although conditioning increased both, pH (from 7.2 to 7.9–8.0) and temperature inside the prism (from ~26 °C to ~34 °C), it did not cause any further changes to chlorophyll content.

### 3.2. Antioxidant Activity

The efficiency of the aqueous WML-P extraction process was 8.2% and was twice lower than that reported by Kim et al. [42]. The discrepancy between the studies was most likely caused by the extraction method (hot plate versus autoclave) and the source of extract (i.e., leaves Polish versus Korean *Morus alba* trees). In previous studies, various extraction methods or extractants and conditions were used [1,34,36,43]. We have previously shown that the best extractant for WML-P is 65% acetone or 65% ethanol [4]. However, in the present study we used only the aqueous extraction process that could be used on semi-technical scale production of dietary supplements and functional foods.

Total phenolics content, measured using Folin-Ciocalteu test, in control WML-P was 2.468 mg GAE/g of dried leaves (Table 2). Conditioning increased phenolic content by 37% (to 3.376 mg GAE/g) and 46% (to 3.591 mg GAE/g) after 2 and 3 h, respectively. Arabshahi-Delouee and Urooj [44] found twice as much total phenolics in aqueous extract of Indian WML. The difference between the studies might be related to differences in extraction temperature (40 °C versus 100 °C).

Compared to results reported by Sánchez-Salcedo et al. [43], WML-P conditioned for 3 or 4 h contained three to four, and control extract five to six times, less polyphenols. However, they [43] used methanol, as an extraction solvent. In this study, we used aqueous extraction in a semi-technical scale production that could contribute to lowering the content of polyphenols.

The antioxidant activity of WML-P was determined using four tests, based on the single electron transfer (SET) mechanism. In the DPPH^•^ test, conditioning for 1 or 2 h increased antiradical activity by 4% to 26%. The ability to quench DPPH^•^ in WML-P was lower than that measured by Iqbal et al. [34] in Spanish WML and more than twice higher than measured in another variety of Polish WML by Tajner-Czopek et al. [45].

In the ABTS^•+^ test we found that conditioning decreased antioxidant activity by up to 32% (after 3 h to 16.033 µM TE/g). Iqbal et al. [34] reported higher ABTS^•+^ values than we found for WML. In the Spanish WML [43] the ABTS^•+^ quenching ability was 10.82–12.00 mg TE/g. In the mentioned earlier study, Tajner-Czopek et al. [45]. Reported three times higher ABTS^•+^ values for aqueous extracts and eight times higher for aqueous-ethanol extracts from leaves of unknown variety and origin. The method of determining antiradical activity using ABTS cation is similar to the DPPH radical method but the test is considered more inclusive. The reason is that ABTS compound is soluble in both aqueous and organic solvents, which ensures a quick reaction with lipophilic and hydrophilic compounds.

The FRAP method allows us to determine the ability to reduce Fe^3+^ from the iron-2,4,6-tripyridyl-S-thiazine (TPTZ) complex by antioxidants present in the sample. Conditioning did not significantly affect the Fe^3+^ reducing ability in WML-P. In the iron chelation test, the conditioning resulted in an increase in chelating ability by 34% (2 h) and 32% (3 h) after conditioning. These results were similar to those reported by Wanyoet et al. in WML from Thai trees [46].

In summary, WML-P conditioning resulted in increased DPPH^•^ quenching capacity, polyphenol content and iron-chelating ability and the highest values were found after 2 and 3 h of conditioning.

### 3.3. Bioactive Compounds

The content of bioactive compounds is presented in Table 3.

The phenolic acids and flavonols belong to the polyphenols and their presence in the raw material largely determines its antioxidant activity. The content of flavonols increased in WML-P extracts after 2 h of conditioning by 70% and then decreased after 3 h and 4 h, although not to the control (0 h) level. Compared to control, phenolic acids content increased after 1 h by 65% from 1.730 to 2.384 mg/g) and 2 h by 38% (to 2.858 mg/g) of conditioning (Table 3A). The predominant phenolic acids were CHL and CAF, which content almost doubled after 2 h of conditioning (from 0.375 to 0.727 mg CHL/g and from 0.378 to 0.726 mg CAF/g). Similar trends were observed for the other phenolic acids. The increase probably was caused by the release of phenolic acids from more complex compounds during conditioning. Among other phenolic acids (GAL, PRO, VAN, FER, SIN) conditioning lowered the CHL and CAF extract concentration. It has been shown that CHL was also dominant in aqueous extracts from other varieties of WML [1,14] and acetone extract from WML-P [6].

Sánchez-Salcedo et al. [43] found CHL content in Spanish WML ranging from 5.29 to7.18 mg/g of dry matter in one study and 12.98 mg/g in another study [46]. In another study, Zhang et al. [47] found CHL in WML from Chinese trees ranging from 1.768 mg/g (Zhejiang, Hangzhou) to 9.617 mg/g (Anhui, Huangshan) in var. Qiangsang1. In this study, GAL and PRO contents in all preparations were similar to those reported in WML from Serbian trees [36]. The increasing amount of CAF and CHL in WML-P indicates that biochemical changes occur during conditioning for 1 and 2 h. Further conditioning causes CAF to break down into caffeic acid and quinic acid. As a result of processing, CHL may be hydrolyzed into two acids, which were quantified in the test sample. The quantitative advantage of caffeic acid over chlorogenic acid in mulberry leaves maybe a marker of the intensity of changes in leaves during processing. It is plausible that the ratio of caffeic acid to chlorogenic acid can be used as a qualitative marker for mulberry leaves.

In the WML-P samples, we identified eight flavonols (Table 3B). The largest amount of flavonols (1.793 mg/g) was found in WML-P conditioned for 2 h. After conditioning for 3 or 4 h, total flavonols content decreased to 1.413 and 1.339 mg/g, respectively) in the total flavonols content. The dominant flavonols were RUT (from 0.283 to 0.580 mg/g), ISQ (from 0.209 to 0.320 mg/g), AST (from 0.203 to 0.435 mg/g), MAL (from 0.157 to 0.274 mg/g). The results were similar to those found in WML from Korean trees [48]. After conditioning for 2 h the RUT content was approximately 100% higher and for 3 or 4 h, approximately 50% higher than in control. This trend may indicate the gradual release of flavonols from bounded forms.

The flavonol quercetin 3-*O*-(6”-*O*-malonyl)-β-d-glucoside (MAL), present in WML, is a bioactive ingredient with antiatherosclerotic and antihyperglycemic capacity. The content of MAL increased by 31% after 2 h of conditioning (to 0.274 mg/g). The further conditioning resulted in a significant reduction in the MAL content (to 0.157 mg/g), while the QUE content increased (to 0.064 mg/g). A plausible explanation is that endogenous esterases released during leaf shredding caused MAL hydrolysis, which resulted in the release of the QUE aglycone. Lee et al. [48] reported that quantitative changes in phenolics content of WML depend on variety, harvest period and heat processing conditions. Similarly, conditioning WML-P for 2 h caused an increase in ISQ (to 0.320 mg/g) and AST (to 0.435 mg/g) content by 53% and 114%, respectively. ISQ was also one of the major flavonols in other studies of WML-P [4,15,49] and in WML from Korean trees [42]. Lee and Choi [48] measured similar amounts of AST.

QUE content in WML-P doubled after conditioning for 4 h, which can be explained by the breakdown of other flavonols containing QUE in their structure, that is, RUT, MAL, ISQ, ISR and was similar to those reported by Kim and Jang [42]. It is speculated that up to 50% of the antidiabetic effect of WML extracts depends of the presence of chlorogenic acid and rutin. Chlorogenic acid can weaken glycogenolysis and reduce glucose uptake and has strong antioxidant properties [1,42]. Rutin protects against cancer and inhibits the peroxidation of LDL cholesterol. The content of rutin in conditioned WML-P was close to those found in WML var. Yun711, Qiangsang1, 7946, Fengtian5 from eastern China [47]. We found that CHL and RUT are present in WML-P in significant quantities. However, CHL can easily disintegrate during processing so that every technological process can change the ratio of caffeic acid to chlorogenic acid.

Another important bioactive component of mulberry leaves is the alkaloid DNJ—1-deoxynojirimycin (Table 3C)—a glucose analogue with a substituted NH group in the pyranose ring with α-glucosidase. This compound has known antioxidant, anti-bacterial and anti-inflammatory inhibitory properties and might also participate in regulating/modulating gut microbiota and having a neuroprotective effect in Alzheimer’s patients [27]. Conditioning did not significantly affect the amounts of DNJ, except for the sample 3 h (to 0.596 mg/g). A similar content of DNJ was found in a few Chinese varieties of WML [47].

In summary, grinding, conditioning and drying caused qualitative and quantitative changes of bioactive components and caused the increased of antioxidant activity of WML samples. These changes were most likely facilitated by enzymes present in the WML such as polyphenol oxidases, hydrolases, synthetases. In addition, the impact of the matrix changes, such as changes in the structural connections within the raw material, including the presence of fiber and its structure, could be also significant. In further studies, the time of conditioning (e.g., 0.5, 1.5, 2.5 h) should be tested and parameters refined. The quantitative relation found between chlorogenic and caffeic acids in products of WML-P may serve as a marker of changes occurring during conditioning of WML-P.

## 4. Conclusions

The conditioning process was successfully utilized in the processing of WML-P. The conditioning process, at ~32–35 °C, caused changes in functional properties and antioxidant activity of WML-P that were time-dependent. Among tested times, conditioning for 1 or 2 h caused the highest increase of the bioactive compounds in the WML-P products. Conditioning of WML-P for 3 and 4 h caused the decrease of the activity of the biologically active WML-P compounds.

## Figures and Tables

**Table 1:** The content of basic compounds, amino acids, minerals, dietary fiber fractions, fatty acids profile and chlorophyll in dried Polish var. zolwinska wielkolistna leaves (WML-P) before (control) and after conditioning.

Component	Derived Products of WML-P				
	0 h (Control)	1 h	2 h	3 h	4 h
**A. Basic Components [g/100 g]**					
Dry matter	99.01 ^a^ ± 0.09	98.95 ^a^ ± 0.08	98.73 ^a^ ± 0.36	98.88 ^a^ ± 0.11	99.09 ^a^ ± 0.02
Ash	15.77 ^e^ ± 0.12	13.29 ^b^ ± 0.05	12.37 ^a^ ± 0.11	13.57 ^c^ ± 0.06	13.83 ^d^ ± 0.03
Lipids	1.79 ^b^ ± 0.16	1.24 ^a,b^ ± 0.47	1.14 ^a^ ± 0.19	1.12 ^a^ ± 0.16	1.02 ^a^ ± 0.03
Proteins	12.78 ^b^ ± 0.33	13.35 ^b^ ± 0.16	12.50 ^a,b^ ± 0.49	13.42 ^b^ ± 0.29	11.50 ^a^ ± 0.73
Carbohydrates	69.67	72.12	73.99	71.88	73.64
**B. Amino Acids (AA) [g/100 g]**					
Phenylalanine (Phe)	1.333 ± 0.044	0.944 ± 0.023	0.957 ± 0.031	1.022 ± 0.053	0.849 ± 0.161
Isoleucine (Ile)	1.126 ± 0.132	0.867 ± 0.038	0.795 ± 0.008	0.895 ± 0.086	0.697 ± 0.098
Leucine (Leu)	2.291 ± 0.177	1.714 ± 0.089	1.588 ± 0.017	1.714 ± 0.040	1.425 ± 0.221
Lysine (Lys)	1.271 ± 0.111	0.920 ± 0.008	0.812 ± 0.022	0.854 ± 0.025	0.686 ± 0.125
Threonine (Thr)	1.353 ± 0.194	1.085 ± 0.051	0.959 ± 0.080	0.945 ± 0.009	0.905 ± 0.127
Valine (Val)	1.431 ± 0.098	1.052 ± 0.062	1.013 ± 0.005	1.132 ± 0.084	0.837 ± 0.124
Arginine (Arg)	2.099 ± 0.162	1.726 ± 0.087	1.521 ± 0.039	1.554 ± 0.033	1.371 ± 0.100
Histidine (His)	0.730 ± 0.021	0.510 ± 0.033	0.477 ± 0.047	0.534 ± 0.026	0.416 ± 0.030
Total EAA	8.805	6.582	6.124	6.562	5.399
Total EAA + semi-EAA	11.634	8.818	8.122	8.650	7.186
Alanine (Ala)	1.883 ± 0.242	1.295 ± 0.093	1.148 ± 0.038	1.213 ± 0.048	1.031 ± 0.115
Glycine (Gly)	1.659 ± 0.073	1.259 ± 0.049	1.298 ± 0.171	1.249 ± 0.006	1.104 ± 0.069
Aspartic acid (Asp)	3.179 ± 0.493	2.584 ± 0.219	2.281 ± 0.093	2.472 ± 0.061	2.100 ± 0.124
Glutamic acid (Glu)	3.605 ± 0.483	2.959 ± 0.428	2.537 ± 0.043	2.733 ± 0.023	2.301 ± 0.303
Proline (Pro)	1.298 ± 0.078	0.947 ± 0.033	0.887 ± 0.032	0.935 ± 0.013	0.766 ± 0.120
Serine (Ser)	1.418 ± 0.116	1.116 ± 0.043	1.038 ± 0.077	1.082 ± 0.006	0.920 ± 0.102
Total NEAA	13.042	10.160	9.189	9.684	8.222
EAA/NEAA	67.5%	64.8%	66.6%	67.8%	65.7%
Total amino acids	24.676	18.978	17.311	18.334	15.408
**C. Fatty Acids (FA) Profile [% of total]**					
Palmitic acid C16:0	27.01 ^a,b,c^ ± 0.65	27.65 ^c^ ± 0.49	27.24 ^b,c^ ± 0.26	25.78 ^a^ ± 0.90	26.23 ^a,b^ ± 0.68
Palmitoleic acid C16:1 n-9 c	2.51 ^a^ ± 0.13	2.39 ^a^ ± 0.46	2.53 ^a^ ± 0.21	2.66 ^a^ ± 0.20	2.61 ^a^ ± 0.21
Margaric acid C17:0	0.66 ^c^ ± 0.01	0.59 ^b,c^ ± 0.10	0.49 ^a,b^ ± 0.06	0,45 ^a^ ± 0.03	0.43 ^a^ ± 0.01
Stearic acid C18:0	6.05 ^a^ ± 0.21	5.16 ^a^ ± 0.30	5.57 ^a^ ± 0.89	4.84 ^a^ ± 0.56	5.55 ^a^ ± 0.63
Elaidic acid C18:1 n-9t	n.d.	n.d.	n.d.	n.d.	n.d.
Oleic acid C18:1 n-9 c	4.46 ^a^ ± 0.38	4.33 ^a^ ± 0.62	4.58 ^a^ ± 0.29	5.41 ^b^ ± 0.24	4.49 ^a^ ± 0.22
Linoleic acid C18:2 n-6 c	10.46 ^a^ ± 0.40	10.94 ^a,b^ ± 0.27	11.68 ^c^ ± 0.26	11.49 ^b,c^± 0.43	11.12 ^a,b,c^ ± 0.14
α-linolenic acid C18:3 n-3	45.60 ^b^ ± 0.57	45.81 ^b^ ± 0.64	44.15 ^a^ ± 0.95	45.66 ^b^ ± 0.31	45.73 ^b^ ± 0.10
Arachidic acid C20:0	0.57 ^a,b^ ± 0.10	0.47 ^a^ ± 0.08	0.66 ^b,c^ ± 0.05	0.73 ^c^ ± 0.02	0.74 ^c^ ± 0.02
Cis-1-eicosenoic acid C20:1	0.55 ^b^ ± 0.13	0.31 ^a^ ± 0.02	0.35 ^a^ ± 0.04	0.30 ^a^ ± 0.03	0.41 ^a^ ± 0.02
Behenic acid C22:0	0.48 ^a^ ± 0.11	0.59 ^a^ ± 0.08	0.59 ^a^ ± 0.06	0.57 ^a^ ± 0.02	0.58 ^a^ ± 0.02
Erucic acid C22:1 n-9	0.72 ^a^ ± 0.11	0.69 ^a^ ± 0.13	1.15 ^b^ ± 0.12	1.08 ^b^ ± 0.11	1.16 ^b^ ± 0.15
Nervonic acid C24:1	0.93 ^a^ ± 0.17	1.07 ^a^ ± 0.03	1.01 ^a^ ± 0.03	1.03 ^a^ ± 0.09	0.95 ^a^ ± 0.03
UFA/SFA	1.87	1.90	1.89	2.09	1.98
**D. Minerals**					
**Macroelements [mg/g]**					
Mg	3.49 ^a^ ± 0.17	3.44 ^a^ ± 0.18	3.37 ^a^ ±0.14	3.54 ^a,b^ ± 0.21	3.51 ^a,b^ ± 0.09
Ca	27.92 ^b^ ± 0.44	25.04 ^a^ ± 0.68	24.72 ^a^ ± 1.63	24.15 ^a^ ± 1.31	25.74 ^a,b^ ± 0.54
K	67.79 ^a^ ± 3.25	64.52 ^a^ ± 2.93	70.96 ^a^ ± 3.17	69.67 ^a^ ± 2.34	64.63 ^a^ ± 2.98
**[µg/g]**					
Na	88.73 ^a^ ± 0.34	95.54 ^b^ ± 0.62	92.99 ^a^ ± 2.25	100.78 ^b^ ± 4.40	94.96 ^a^ ± 6.11
**Microelements [µg/g]**					
Fe	79.87 ^a^ ± 1.11	79.73 ^a^ ± 8.69	78.74 ^a^ ± 1.15	78.30 ^a^ ± 5.33	80.28 ^a^ ± 7.75
Mn	27.01 ^a^ ± 1.33	27.11 ^a^ ± 1.77	26.81 ^a^ ± 0.35	27.54 ^a^ ± 1.09	25.03 ^a^ ± 2.18
Zn	24.09 ^a^ ± 0.74	23.57 ^a^ ± 0.69	24.20 ^a^ ± 0.79	25.54 ^a^ ± 1.38	24.14 ^a^ ± 1.04
Cu	4.98 ^a^ ± 0.89	5.22 ^a^ ± 0.35	5.02 ^a^ ± 0.26	4.95 ^a^ ± 0.32	5.09 ^a^ ± 0.69
**E. Dietary Fiber and Fractions [g/100 g]**					
NDF	26.42 ^a^ ± 1.16	27.91 ^a,b^ ± 0.98	27.83 ^a,b^ ± 0.56	30.01 ^b^ ± 0.36	29.73 ^b^ ± 1.07
ADF	19.34 ^a^ ± 0.40	20.06 ^a^ ± 0.45	20.30 ^a^ ± 1.01	20.67 ^a^ ± 0.98	19.92 ^a^ ± 0.78
ADL	0.10 ^a^ ± 0.01	0.09 ^a^ ± 0.01	0.13 ^b^ ± 0.01	0.18 ^c^ ± 0.01	0.16 ^c^ ± 0.00
Hemicelluloses	7.09 ^a^ ± 0.98	7.84 ^a,b,c^ ± 0.91	7.53 ^a,b^ ± 0.73	9.33 ^b,c^ ± 0.69	9.81 ^c^ ± 0.48
Celluloses	19.23 ^a^ ± 0.40	19.97 ^a^ ± 0.45	20.17 ^a^ ± 1.00	20.49 ^a^ ± 0.98	19.76 ^a^ ± 0.78
IDF	54.57 ^a^ ± 0.91	58.57 ^b^ ± 0.47	58.61 ^b^ ± 1.29	59.75 ^b^ ± 1.24	58.74 ^b^ ± 0.47
SDF	4.44 ^a^ ± 0.13	4.49 ^a^ ± 0.24	4.91 ^a^ ± 0.17	4.47 ^a^ ± 0.42	4.44 ^a^ ± 0.23
TDF	59.01 ^a^ ± 0.84	63.06 ^b^ ± 0.70	63.52 ^b^ ± 1.12	64.23 ^b^ ± 1.43	63.18 ^b^ ± 0.46
**F. Chlorophyll [mg/g]**					
Chlorophyll a	1.834 ^b^ ± 0.023	1.216 ^a^ ± 0.010	0.945 ^a^ ± 0.064	1.121 ^a^ ± 0.297	1.217 ^a^ ± 0.135
	66%	59%	61%	60%	55%
Chlorophyll b	0.919 ^a^ ± 0.082	0.834 ^a^ ± 0.172	0.602 ^a^ ± 0.140	0.747 ^a^ ± 0.312	1.015 ^a^ ± 0.405
	34%	41%	39%	40%	45%
Total	2.753	2.050	1.546	1.868	2.232

0 h—control leaves; 1 h—leaves conditioned for 1 h; 2 h—leaves conditioned for 2 h; 3 h—leaves conditioned for 3 h; 4 h—leaves conditioned for 4 h; Total EAA—total essential amino acids; Total NEAA—total non-essential amino acids; semi–EAA—semi–essential amino acids; UFA—unsaturated fatty acids; SFA—saturated fatty acids; NDF—neutral dietary fiber; ADF—acid detergent fiber; ADL—detergent lignin; IDF—insoluble dietary fiber; SDF—soluble dietary fiber; TDF—total dietary fiber; chlorophyll %—percentage fraction in total chlorophyll; ^a, b, c, d, e^—different letters show statistically significant differences in Tukey’s test (α < 0.05).

**Table 2:** Antioxidant activity of dried Polish var. zolwinska wielkolistna leaves (WML-P) before (control) and after conditioning.

Test	Derived Products of WML-P				
	0 h (Control)	1 h	2 h	3 h	4 h
Total Phenolics [mg GAE/g]	2.468 ^a^ ± 0.500	2.352 ^a^ ± 0.074	3.376 ^b^ ± 0.034	3.591 ^b^ ± 0.028	2.175 ^a^ ± 0.067
ABTS^•+^ Test [µM TE/g]	23.630 ^d^ ± 0.019	23.892 ^d^ ± 0.004	17.235 ^b^ ± 0.011	16.033 ^a^ ± 0.004	20.110 ^c^ ± 0.003
DPPH^•^ Test [µM TE/g]	49.420 ^a^ ± 0.005	53.241 ^c^ ± 0.014	62.517 ^d^ ± 0.004	51.323 ^b^ ± 0.009	51.546 ^b^ ± 0.052
Chelating Activity [%]	40.383 ^a^ ± 0.164	53.174 ^d^ ± 0.388	54.129 ^d^ ± 0.842	50.471 ^c^ ± 0.827	43.085 ^b^ ± 0.623
FRAP Test [µM Fe^2+^/mg]	0.221 ^a^ ± 0.042	0.248 ^a^ ± 0.034	0.250 ^a^ ± 0.013	0.187 ^a^ ± 0.023	0.190 ^a^ ± 0.042

0 h—control leaves; 1 h—leaves conditioned for 1 h; 2 h—leaves conditioned for 2 h; 3 h—leaves conditioned for 3 h; 4 h—leaves conditioned for 4 h; ^a, b, c, d^—different letters show statistically significant differences in Tukey’s test (α < 0.05).

**Table 3:** The content of bioactive compounds in dried Polish var. zolwinska wielkolistna leaves (WML-P) before (control) and after conditioning.

Bioactive Compounds	Derived Products of WML-P				
	0 h (Control)	1 h	2 h	3 h	4 h
**A. Phenolic Acids [mg/g]**					
Gallic Acid (GAL)	0.190 ^a^ ± 0.005	0.268 ^c^ ± 0.001	0.261 ^c^ ± 0.007	0.231 ^b^ ± 0.006	0.197 ^a^ ± 0.001
Protocatechuic Acid (PRO)	0.137 ^a^ ± 0.009	0.256 ^d^ ± 0.002	0.259 ^d^ ± 0.004	0.181 ^b^ ± 0.012	0.215 ^c^ ± 0.003
4-hydroxybenzoic Acid (HYD)	0.100 ^a^ ± 0.001	0.111 ^a,b^ ± 0.001	0.119 ^b^ ± 0.003	0.112 ^a,b^ ± 0.010	0.125 ^b^ ± 0.005
Vanillic Acid (VAN)	0.160 ^a^ ± 0.003	0.219 ^b^ ± 0.002	0.234 ^b,c^ ± 0.002	0.245 ^c^ ± 0.015	0.311 ^d^ ± 0.004
Chlorogenic Acid (CAF)	0.375 ^b^ ± 0.003	0.528 ^c^ ± 0.012	0.727 ^d^ ± 0.015	0.358 ^b^ ± 0.017	0.162 ^a^ ± 0.011
Caffeic Acid (CHL)	0.378 ^b^ ± 0.007	0.527 ^c^ ± 0.012	0.726 ^d^ ± 0.015	0.347 ^b^ ± 0.018	0.164 ^a^ ± 0.012
*p*-coumaric Acid (CUM)	0.047 ^a^ ± 0.007	0.065 ^a^ ± 0.003	0.061 ^a^ ± 0.012	0.159 ^b^ ± 0.068	0.098 ^a,b^ ± 0.003
Ferullic Acid (FER)	0.139 ^a^ ± 0.006	0.231 ^c^ ± 0.005	0.265 ^d^ ± 0.009	0.200 ^b^ ± 0.007	0.160 ^a^ ± 0.013
Sinapic Acid (SIN)	0.204 ^b^ ± 0.012	0.179 ^a^ ± 0.008	0.206 ^b^ ± 0.004	0.277 ^c^ ± 0.007	0.309 ^d^ ± 0.004
Total	1.730	2.384	2.858	2.110	1.741
**B. Flavonols [mg/g]**					
Rutin (RUT)	0.283 ^a^ ± 0.014	0.550 ^c^ ± 0.049	0.580 ^c^ ± 0.001	0.429 ^b^ ± 0.021	0.421 ^b^ ± 0.075
Isoquercetin (ISQ)	0.209 ^a^ ± 0.009	0.283 ^b^ ± 0.011	0.320 ^c^ ± 0.001	0.283 ^b^ ± 0.006	0.287 ^b^ ± 0.018
Quercetin 3-*O*-(6′′-*O*-malonyl)-β-d-glucoside (MAL)	0.207 ^b^ ± 0.025	0.258 ^c^ ± 0.033	0.274 ^c^ ± 0.009	0.206 ^b^ ± 0.010	0.157 ^a^ ± 0.004
Astragalin (AST)	0.203 ^a^ ± 0.012	0.342 ^b^ ± 0.011	0.435 ^c^ ± 0.004	0.321 ^b^ ± 0.044	0.313 ^b^ ± 0.078
Myricetin (MYR)	0.062 ^a^ ± 0.001	0.145 ^d^ ± 0.011	0.156 ^e^ ± 0.001	0.119 ^c^ ± 0.002	0.089 ^b^ ± 0.002
Quercetin (QUE)	0.032 ^b^ ± 0.005	0.022 ^a^ ± 0.004	0.025 ^a,b^ ± 0.000	0.049 ^c^ ± 0.009	0.064 ^d^ ± 0.000
Kaempferol (KEM)	0.008 ^c^ ± 0.001	0.004 ^a,b^ ± 0.001	0.003 ^a^ ± 0.000	0.004 ^b^ ± 0.000	0.008 ^c^ ± 0.000
Isorhamnetin (ISR)	0.001 ^b^ ± 0.000	0.001 ^b^ ± 0.000	0.000 ^a^ ± 0.000	0.002 ^c^ ± 0.000	0.000 ^a^ ± 0.000
Total	1.005	1.605	1.793	1.413	1.339
**C. Iminosugar [mg/g]**					
1-deoxynojirimycin (DNJ)	0.617 ^b^ ± 0.038	0.472 ^a^ ± 0.017	0.461 ^a^ ± 0.030	0.596 ^b^ ± 0.017	0.394 ^a^ ± 0.058

0 h—control leaves; 1 h—leaves conditioned for 1 h; 2 h—leaves conditioned for 2 h; 3 h—leaves conditioned for 3 h; 4 h—leaves conditioned for 4 h; ^a, b, c, d, e^—different letters show statistically significant differences in Tukey’s test (α < 0.05).
